# What Data Should Be Included in a Modern Public Health Data System

**DOI:** 10.1089/big.2022.0205

**Published:** 2022-09-06

**Authors:** Joie D. Acosta, Anita Chandra, Douglas Yeung, Christopher Nelson, Nabeel Qureshi, Tara Blagg, Laurie T. Martin

**Affiliations:** ^1^ RAND Corporation, Arlington, Virginia, USA.; ^2^ RAND Corporation, Santa Monica, California, USA.; ^3^ Pardee RAND Graduate School, Santa Monica, California, USA.

**Keywords:** equity, public health, measures, well-being

## Abstract

The public is inundated with data, both in where data are ubiquitously collected and in how organizations are using data to drive public sector and commercial decisions. The public health data system is no exception to this flood of data, both in growing data volume and variety. However, what are collected and analyzed about the health status of the nation, how particular data and measures are prioritized for parsimony, and how those data provide a signal for where to invest to address health inequities are in dire need of a reboot. As with other articles in this supplement, this article builds from a literature review, an environmental scan, and deliberations from the National Commission to Transform Public Health Data Systems. The article summarizes what data should be included and identifies where the technology and data sectors can contribute to fill current gaps to measure equity, positive health, and well-being.

## Introduction

It can be argued that there is a significant volume of data on health and well-being, but that these data are not providing the information needed to fully advance effective and equitable public health action today. The role of equity is central to a modern public health data system, and the recognition that the current data system falls short in that objective is clear. However, there are other interrelated challenges to identifying and tracking populations affected by multiple stresses, such as COVID-19 and chronic disease. There is an opportunity to reimagine what matters for health in the United States; how the nation uses data as a tool to aid action on inequity; and how the United States uses public health data to capture concepts, such as systemic health injustice, well-being, and resilience.

In this article, we describe the types and content of data needed in this reimagined public health data system. Advancing the culture of health, one that is guided by the cultural and social drivers of health, requires a different approach to public health data collection and use, including how to move beyond a focus on illness and capture concepts such as positive health and well-being.^1^ To date, public health data have not always led to appropriately targeted public health action, in large part, because there is no integrated or coordinated public health data system to support sense-making (described in the prior article) and data-informed decision-making. As with the move to an equity-centered public health data system, the opportunities to pool new and different types of data provide a window for many sectors, outside formal public health, to engage in new ways.

With access to a greater variety of data and potentially faster use of data, the data science and technology sector has an important role to play.

## Methods

In 2020, the Robert Wood Johnson Foundation formed the National Commission to Transform Public Health Data Systems to review significant challenges to the current public health data system, and “provide recommendations to policymakers, health care organizations and institutions, service providers, and philanthropy” on potential solutions to overcome these challenges.^2^

In support of this effort, RAND conducted a supporting analysis that included an environmental scan to identify key issues, points of consideration, trade-offs, and tensions, and current activities related to public health data, data systems, and data modernization efforts. This effort included a targeted scan of published research articles and reports, reviews of websites and working documents describing coordinated activities (e.g., data interoperability), and recent initiatives. Additional searches included the use of “big data” in public health, data privacy, and ethics of public health data collection. Although the team primarily focused on public health data, it also identified seminal articles and reports from other sectors or disciplines whose findings could apply to public health data systems.

RAND simultaneously conducted semistructured interviews with 112 experts and thought leaders on the main topics before the Commission. Individuals represented diverse sectors, including public health and health care, technology and data science, research and policy, journalism, and law. The interviews also included experts in data, data use, equity, community engagement, and research translation who work *outside* the traditional health sector. The project was reviewed and approved by the RAND Human Subjects Protection Committee.

In this article, we highlight relevant findings from this supporting analysis and then implications for the data science and technology sectors, with consideration of recommendations that emerged from the final Commission report.

## Findings

Four major themes emerged from the supporting analysis regarding the approach to collecting and reporting public health data; the content of public health data; the volume and coordination of data across sectors, particularly unstructured data; and the level of precision, granularity, and timeliness of data currently reflected in public health data sources.^3^ These themes were further affirmed in the Commission recommendations related to ensuring public health data include information on structural racism and other drivers of inequity.^2^ In the following section, we briefly describe these four themes.

### The modern public health data system is reactive rather than proactive and covers a wide array of measures rather than a smaller prioritized set

The main function of public health data has remained consistent over the years, primarily focused on population health surveillance, one of the 10 essential public health services.^†,4^ However, data are used to support other public health services, including the selection of public health actions, communication with the public, and the building of partnerships to improve health. Despite these varied uses of public health data, the content of the data has been augmented in ways that are more often reactive in monitoring health conditions.^3^ This often includes the disproportionate use of lagging indicators (meaning data on performance today or what *has* occurred), rather than leading indicators that portend future health needs. Data systems have been enhanced as the nation has learned more about key factors that influence health.

Healthy People, the national strategic management plan that guides health promotion and disease prevention, demonstrates the steady expansion of public health's primary areas of focus over the past 40 years.^5^ This focus has expanded from a singular emphasis on mortality to now include aspects of quality of life, health disparities and health equity, and social and physical environments. Yet, despite the expansion in these focus areas, the data and indicators that are often a part of public health data emphasize infectious disease, chronic conditions, mortality, and risk factor exposures, with less attention to other factors that influence health over generations (e.g., trauma), as well as measures of positive health and well-being and systemic inequities.

In addition to the need for data that can support proactive response is the need for narrowing the data that are used to facilitate that response. Modernizing the public health data system requires prioritizing a smaller set of core national measures to ensure the parsimony needed to proactively make social change on a few key public health efforts, rather than continue to spread the primary focus of public health thin across many areas.^3^ If addressing systemic health injustice becomes the priority, then the primary areas of focus and the associated measures and indicators should emphasize upstream drivers of inequity before those inequities become disparities.

However, the current use of the public health data system in the United States does not proportionately weight measures toward any clearly operationalized priority, whether positive health, addressing inequity, or other forward-leading public health action. As such, the current public health data system does not fully communicate a common set of values through an alignment and parsimony of national measures.

### The content of public health data misses important information about structural inequity and positive health and well-being

Even with increasing amounts of health data being collected, public health data are limited in their ability to inform decision-making because data on many upstream factors that contribute to health are not regularly or consistently collected. A review of the Healthy People 2030 Leading Health Indicators by social determinants of health suggests continued gaps in current public health data, particularly related to economic stability, neighborhood and the built environment, education, and community and social context.^6^ A broader review of national public health data sources conducted for this supporting analysis revealed similar gaps.^3^

There are several U.S. efforts underway that are working to include broader conceptualizations of well-being in surveys. This includes the Gallup survey, which captures subjective well-being (e.g., optimism, hope, resilience) and happiness^7^; Measure of America, which includes some sentinel measures of well-being and equity^8^; and Well-Being in the Nation, which includes measures of well-being and has advanced the consistent use of the Cantril ladder, a measure of life evaluation and expectation linked to other measures of mental health and well-being in a population.^9^ Healthy People 2030 has begun to integrate subjective well-being and to push those measures in national surveys, such as the Behavior Risk Factor Surveillance System.

The Centers for Disease Control and Prevention has integrated well-being concepts for some time, yet currently those are mostly linked to Health-Related Quality of Life.^10^ However, areas such as lifelong learning and prosocial health behaviors, useful constructs for understanding drivers of health outcomes, are noticeably absent even from these efforts to advance well-being assessment. Although there has been advancement in the use of surveys to capture subjective well-being and understand the drivers of well-being, there are opportunities to use more information coming from the private sector to fill these gaps and consistently link information on sentiment and community needs to inform public health action. To date, these efforts are not standardized.^11^

### Handling the volume of new public health data requires greater support and coordination, particularly for unstructured data

Unstructured data refers to nonstandardized data from a variety of sources (e.g., social media, clouds, sensors) and can include text, images, audio, video, blogs, websites, and so forth.^12–16^ Unstructured data are growing exponentially, with more than 2.5 quintillion bytes of unstructured data generated every day. Unstructured data are expected to continue to grow over time, with an estimated 65 percent annual growth rate, and to comprise ∼95 percent of global data.^14^ These unstructured data can provide critical insights into public health; however, these data have not yet been effectively integrated into public health data systems. For example, clinical notes and other free text from electronic health records have been analyzed for insights into patient care and overprescribing and use of certain medications.^17^

Text from social media posts has been analyzed to look for a positive or a negative sentiment providing insight into the psychological well-being of a population, as well as self-reported symptoms of a disease to help estimate disease outbreak.^18,19^ There is emerging research about the use of images posted on social media to track drinking, smoking, and obesity-related behaviors and the use of wearable sensors for remote patient monitoring and virtual health assessments.^20,21^ The huge volume and complexity of existing and unstructured data make it a tedious task to extract useful information from different types of data, and its use is fraught with concerns about ethical trade-offs. More data are not useful unless they can be used for proactive decisions.

While past research has demonstrated how unstructured data can inform public health decision-making, these investigations have raised concerns about how to maintain privacy, especially if artificial intelligence is used to generate or distill information that could be considered personally identifiable medical information.^22^ Data ownership is another ethical challenge identified in using unstructured data and which will require strong governance policies.

### Precision, granularity, and timeliness of public health data continue to challenge the ability to make responsive decisions

The types of public health data have adapted according to the changing public health data and informatic needs, moving from counts and trends to causal inferences to geospatial inferences over time.^23^ Public health practitioners often rely on federal data systems to create those inferences, but those data must be collected, cleaned, and deidentified, creating significant time lags often measured in years. This requires significant resources and can hinder public health departments in making timely decisions because they are constantly relying on survey data that may be lagging by a year or more.

Making key decisions with outdated information can negatively impact public health, particularly if populations are dealing with a public health emergency.^24^ For example, the lack of timely, federal data made it more difficult to inform a pandemic containment strategy and shed new light on the limitations of our current public health data system for supporting real-time decision-making. While countries such as South Korea, Singapore, and New Zealand were disseminating public health data in near real-time, the U.S. data lagged and were often lacking critical information, such as race and ethnicity.^25^

## Implications

The stakeholder analysis, review of existing data systems, and Commission deliberations identified key areas in which the data science and technology sector can be particularly useful.^2,3^ For example, one of the Commission's recommendations was focused on shifting the national health narrative to one that is equity-based and positively oriented.^2^ As noted in the findings described earlier, there is a large gap in the data available (both in time and quality) on positive health, well-being, and the drivers of structural inequity, some of which could be informed by the data science and technology sector specifically. Furthermore, data science and technology experts often have greater understanding of how to handle the dimensions of “big data” in terms of data structure, precision, and data volume. We further describe those two key implications for this sector with respect to the content of public health data.

### The technology sector has information that can support public health action regarding positive health, well-being, and structural inequity

Many companies are examining the data they collect and own and are seeking to repurpose those data for public good.^26,27^ For instance, person-level, transactional data exist on almost every aspect of our lives and are available to private industry. Technologies, such as global positioning system and accelerometry data from wearable devices, provide insight into bodily movements and location. Purchasing and travel activity provide insight on personal values, socioeconomic status, and healthy behaviors. Online habits, such as web searches or browsing histories, can illuminate what people want to know about health, as well as offer sensitive markers of health status.^28^ Because of network effects or first mover effects (i.e., the benefits accrued from being the first to market), much of the data for a given technology type are monopolized by a single dominant company in that domain.

Technology companies that seek to leverage their own products and holdings for social good are largely doing it on their own—that is, looking inward to their own data and tools and focusing on their own capabilities. One way in which some companies (e.g., Twitter) have tried to do this is by making subsets of their data openly available for researchers or government use, under specific data use or nondisclosure agreements. However, these data tend to be scrubbed of personal identifying information and limited in scope or by region, which may be necessary for privacy and data security, but can limit their usefulness for public health action. Currently, public health does not have a consistent, established relationship with technology companies to obtain or leverage the data or innovative analysis of unstructured and “big data” that many of these companies are undertaking. However, a few notable exceptions provide promising models for such collaborations.

### The data science and technology sector can offer approaches for balancing precision and granularity with real-time response

Existing public health data challenges related to the speed and precision trade-off may be overcome by learning from and partnering with technology companies, which have resources and data capabilities that, in many cases, far exceed the typical public health sector. These companies have data that are already continually collected and analyzed. Using massive amounts of data in combination with sophisticated analytic techniques, companies can create accurate profiles of individuals or population segments based on numerous characteristics. These capabilities can be used to rapidly identify behavior changes, to answer emerging questions, or to spot shifts in sentiment. Social media posts or web searches provide real-time granular information that can reveal mental states such as depression or anxiety, or whether someone has searched for information about how to hurt themselves or others.^29^

Many products that collect data are already in our households, which could help lessen a reliance on traditional data collection procedures that are more laborious and take longer to implement. One impact of this could be to increase the representation in public health data of an otherwise-hidden population.^30,31^ Attention should still be paid to individuals who may not be online due to the lack of broadband in some regions, limited access to free public internet, inconsistent access to the internet due to economic instability, and varying degrees of digital literacy. It will be critical to acknowledge and address this digital divide to not create further inequity among populations that bear significant public health burdens but are not reflected in online data.^32^

## Conclusion

The content of current public health data for proactive public health action and equity-centeredness is insufficient. The data science and technology sector has an important, although still largely untapped, role in supporting how public health captures more temporally relevant and actionable data. For the relationship between formal public health and the technology sector to leverage the new window for public health data system modernization, the two sectors will need to have open and consistent dialogue about the content of available data, its utility for certain types of public health action, and the governance required to support ethical and equitable application.

## Notes

^†^
Ten essential public health services: (1) Assess and monitor population health status, factors that influence health and community needs and assets. (2) Investigate, diagnose, and address health problems and hazards affecting the populations. (3) Communicate effectively to inform and educate people about health, factors that influence it, and how to improve it. (4) Strengthen, support, and mobilize communities and partnerships to improve health. (5) Create, champion, and implement policies, plans, and laws that impact health. (6) Utilize legal and regulatory actions designed to improve and protect the public's health. (7) Assure an effective system that enables equitable access to the individual services and care they need to be healthy. 8. Build and support a diverse and skilled public health workforce. (9) Improve and innovate public health functions through ongoing evaluation, research, and continuous quality improvement. (10) Build and maintain a strong organizational infrastructure for public health.
